# Haemoglobinopathies and other rare anemias in Spain: ten years of a nationwide registry (REHem-AR)

**DOI:** 10.1007/s00277-024-05788-8

**Published:** 2024-05-20

**Authors:** José Manuel Marco Sánchez, Eduardo Jesús Bardón Cancho, David Benéitez, Salvador Payán-Pernía, Anna Collado Gimbert, Anna Ruiz-Llobet, José Antonio Salinas, Elena Sebastián, Bienvenida Argilés, Mar Bermúdez, María Ángeles Vázquez, María José Ortega, Montserrat López Rubio, Ainhoa Gondra, José Javier Uriz, Marta Morado, María Teresa Coll, Mónica López Duarte, María Baro, Áurea Cervera, Valle Recasens, Carmen García Blanes, María Pozo del Carcavilla, María Tallon, Ana González Espín, Filip Camil Olteanu Olteanu, Pablo González, María Mar del Mañú Pereira, Elena Cela

**Affiliations:** 1 Data Manager of the Spanish Registry of Rare Haemoglobinopathies and Rare Anaemias (REHem-AR), Gregorio Marañón Health Research Institute. Section of Pediatric Hemato-Oncology.Pediatrics Service, Hospital General Universitario Gregorio Marañón, O’Donnell, 48, Madrid, Spain; 2 CSUR Erithropathology. ERN-EuroBloodNet. CIBERER, Universidad Complutense de Madrid, Madrid, Spain; 3 Section of Pediatric Hemato-Oncology. Pediatrics Service. Hospital General, Universitario Gregorio Marañón, O’Donnell, 48, Madrid, Spain; 4 Hematology Service. Hospital Universitario Vall d’Hebron. Barcelona. ERN-Eurobloodnet, Universitat Autònoma de Barcelona, Passeig de La Vall d’Hebron, 119-129 Barcelona, Spain; 5 Hematology Service. Hospital Universitario Virgen del Rocío, Instituto de Biomedicina de Sevilla (IBiS), Av. Manuel Siurot, S/N, 41013 Seville, Spain; 6 Section of Pediatric Hemato-Oncology. Pediatrics Service, Hospital Universitario Vall d’Hebron. Barcelona, Passeig de La Vall d’Hebron, 119-129 Barcelona, Spain; 7 Hematology Service. Hospital Sant Joan de DéuUniversitat de Barcelona. Institut de Recerca Hospital Sant Joan de Déu. CSUR Eritropatología. ERN-EuroBloodNet, Passeig de Sant Joan de Déu, 2Esplugues de Llobregat, Barcelona, Spain; 8 Section of Pediatric Hemato-Oncology. Pediatrics Service, Hospital Universitari Son Espases, Carretera de Valldemossa, 79, 07120 Palma, Illes Balears Spain; 9 Section of Pediatric Hemato-Oncology. Hospital Infantil Universitario Niño Jesús. Foundation for Biomedical Research of the Niño Jesús University Childrens Hospital, Av. de Menéndez Pelayo, 65, 28009 Madrid, Spain; 10 Section of Pediatric Hemato-Oncology. Pediatrics Service. Hospital, Universitario y Politécnico La Fe, Avinguda de Fernando Abril Martorell, 106, Valencia, Spain; 11 Section of Pediatric Hemato-Oncology. Pediatrics Service. Hospital Clínico, Universitario Virgen de La Arrixaca, Ctra. Madrid-Cartagena, S/N, 30120El Palmar, Murcia, Spain; 12 Section of Pediatric Hematology. Hospital Materno-Infantil Torrecárdenas, Calle Hermandad de Donantes Sangre S/N, 04009 Almería, Spain; 13 Section of Pediatric Hematology. Hospital, Universitario Virgen de Las Nieves, Av. de Las Fuerzas Armadas, 2, 18014 Granada, Spain; 14 Hematology Service. Hospital Universitario Príncipe de Asturias, Carretera de Alcalá Meco S/N, 28805 Alcalá de Henares, Madrid Spain; 15 Section of Pediatric Hemato-Oncology. Pediatrics Service, Hospital Universitario de Basurto, Universidad del País Vasco UPV/EHU, Montevideo Etorb, 18, 48013 Bilbao, Bizkaia Spain; 16 Section of Pediatric Hemato-Oncology. Pediatrics Service, Hospital Universitario Donostia, , Begiristain Doktorea Pasealekua, S/N, 20014 Donostia, Gipuzkoa Spain; 17 Hematology Service. Hospital Universitario La Paz, Paseo de La Castellana 261, 28046 , Madrid, Spain; 18 Section of Pediatric Hemato-Oncology. Pediatrics Service, Hospital General de Granollers. , Carrer de Francesc Ribas, S/N, 08402 Barcelona, Granollers Spain; 19 Hematology Service, Hospital Universitario Marqués de Valdecilla, Av Valdecilla S/N, 39008 Santander, Cantabria Spain; 20 Section of Pediatric Hemato-Oncology. Pediatrics Service, Hospital Doce de Octubre, Avenida Córdoba S/n2, 28041 Madrid, Spain; 21 Pediatric Service, Hospital Universitario Móstoles, C. Dr. Luis Montes, S/N, 28935 Madrid, Móstoles Spain; 22 Hematology Service. Hospital Miguel Servet, P.º de Isabel La Católica, 1-3, 50009, Zaragoza, Spain; 23 Section of Pediatric Hemato-Oncology. Pediatrics Service, Hospital Clínico Valencia, Av Blasco Ibáñez, 17, 46010 Valencia, Spain; 24 Section of Pediatric Hemato-Oncology. Pediatrics Service, Complejo Hospitalario Albacete, C. Hermano Falco, 37, 02006 Albacete, Spain; 25 Section of Pediatric Hemato-Oncology. Pediatrics Service, Hospital Álvaro Cunqueiro, Estrada de Clara Campoamor, 341, 36312 Vigo, Spain; 26 Section of Pediatric Hemato-Oncology. Pediatrics Service, Complejo Hospitalario Jaén, Av Ejército Español, 10, 23007 Jaén, Spain; 27 Section of Pediatric Hemato-Oncology. Pediatrics Service. Hospital, Universitario de Guadalajara, C Donante de Sangre, S/N, 19002 Guadalajara, Spain; 28 Hospital General Universitario Gregorio Marañón, Calle O’Donnell, 48, Madrid, Spain; 29 Hospital Universitario Vall d’Hebron. BarcelonaERN-EurobloodnetUniversitat Autònoma de Barcelona, Passeig de La Vall d’Hebron, 119-129 Barcelona, Spain; 30 Coordinator of REHem-AR. Erythropathology Working Group of the Spanish Society of Pediatric Hematology and Oncology (SEHOP), Hospital General Universitario Gregorio Marañón, Madrid, Spain

**Keywords:** Hemoglobinopathies, Rare anaemias, Nationwide registry

## Abstract

REHem-AR was created in 2013. The progressive implementation of neonatal screening for haemoglobinopathies in Spanish autonomous communities where the registry had not been implemented, as well as the addition of new centres during this period, has considerably increased the sample of patients covered. In this study, we update our previous publication in this area, after a follow-up of more than 5 years. An observational, descriptive, multicentre and ambispective study of adult and paediatric patients with haemoglobinopathies and rare anaemias registered in REHem was performed. The data are from a cross-sectional analysis performed on 1 June, 2023. The study population comprised 1,756 patients, of whom 1,317 had SCD, 214 had thalassaemia and 224 were diagnosed with another condition. Slightly more than one third of SCD patients (37%) were diagnosed based on neonatal bloodspot screening, and the mean age at diagnosis was 2.5 years; 71% of thalassaemia patients were diagnosed based on the presence of anaemia. Vaso-occlusive crisis and acute chest syndrome continue to be the most frequent complications in SCD. HSCT was performed in 83 patients with SCD and in 50 patients with thalassaemia. Since the previous publication, REHem-AR has grown in size by more than 500 cases. SCD and TM are less frequent in Spain than in other European countries, although the data show that rare anaemias are frequent within rare diseases. REHem-AR constitutes an important structure for following the natural history of rare anaemias and enables us to calculate investment needs for current and future treatments.

## Introduction

Haemoglobinopathies are a clinically heterogeneous group of inherited diseases caused mainly by mutations and/or deletions in globin genes. The most clinically significant mutations are those affecting α and β [1] globins. Haemoglobinopathies are classified into thalassaemic haemoglobinopathies, or thalassaemias, in which there is a deficit in the synthesis of the globin affected, and structural haemoglobinopathies, in which there are changes in the amino acid sequence and whose main subgroup is characterised by haemoglobin S, or sickle cell disease (SCD). Depending on the mutation acquired, the other structural haemoglobinopathies identified include haemoglobin C, D-Punjab and O-Arab disease. We can also find mixed forms that combine characteristics of both groups, such as haemoglobin E or haemoglobin Lepore trait. The pathophysiology and clinical pattern of each disease can be very broad, as reported elsewhere [2].

The annual number of newborns with SCD, estimated at 305,800 worldwide in 2010, is likely to increase by about one third by 2050 [3–5].

In Madrid, the neonatal prevalence of haemoglobinopathy is 5.57 per 1,000 births; in the case of SCD, it is 1 per 6,250 births [6]. In other countries, such as the UK, between 12,500 and 15,000 people are estimated to have SCD [7].

Since the creation in 2013 of the Spanish Registry of Haemoglobinopathies and Rare Anaemias (REHem-AR), the demographic and clinical characteristics of haemoglobinopathies have been recorded. The latest publication on this subject dates from 2019, with data updated up to 31 December 2017 [6]. The progressive implementation of neonatal screening for SCD throughout Spain, as well as the inclusion of new centres in the registry during this period, has considerably increased the sample of patients. In the present study, we update the data provided by the registry, after a follow-up of more than 5 years.

## Material and methods

The REHem-AR, which was launched in January 2013 and is monitored annually, collects data on patients with clinically significant rare anaemias in Spain, including transfusion-dependent thalassaemias (TDT), non-transfusion-dependent thalassaemias (NTDT), SCD and other clinically significant haemoglobinopathies, as well as enzymopathies.

This observational, descriptive, multicentre and ambispective study includes adult and paediatric patients with haemoglobinopathies and other rare anaemias registered in REHem-AR. The registry collected patients' clinical data retrospectively at the date of creation in 2013. New patients are registered and followed prospectively if they are registered at birth, and retrospectively if they are registered at an older age. Patient information is updated on an annual basis.

The data presented in the present study are from a cross-sectional analysis performed on 1 June 2023 and update those reported in the previous 2017 publication. The study population comprises patients of any age who made at least 1 visit to any paediatric or adult haematology unit in Spain. Inclusion in the cohort was at birth if neonatal screening was available or at the date of diagnosis if performed later.

Informed consent was obtained from all patients or their legal guardians in accordance with the Declaration of Helsinki. For patients who died or were lost to follow-up before initiation of the study, data were collected retrospectively from the corresponding medical records.

The registry and the data analysis were approved by the Ethics Committee of each participating centre, by the Office of the Public Prosecutor for Minors and by the Spanish Data Protection Agency. The Spanish Agency of Medicines and Medical Devices was informed of our intention to perform the study, which was sponsored by the Spanish Society of Paediatric Haematology and Oncology (SEHOP).

The different variables were entered online by each of the treating physicians and are expressed as median and interquartile range. The items entered included identification data, date of birth, sex, diagnosis and date of diagnosis, reason for diagnosis, country of birth, genotype, imaging test results, clinical complications, treatments and follow-up data (alive or deceased, lost to follow-up and causes). Potentially duplicate records were examined and excluded based on the patient identification code of the National Health System, when available. In certain cases, the combination of the patient’s name, sex, date of birth and personal contact with the treating physician was necessary to identify duplicates.

### Definition of variables

SCD comprises a group of chronic haemoglobinopathies characterised by haemolysis and intermittent episodes of vascular occlusion causing tissue ischaemia and acute and chronic organ dysfunction. It is a genetic, autosomal recessive disease defined by the presence of haemoglobin S (HbS) in erythrocytes, and our registry contains homozygous or compound heterozygous individuals with several possible genotypes, namely, SS, SC, Sβ^+^, Sβ^0^ and SD [8].

Thalassaemias result from decreased or absent synthesis of 1 or more globin chains that are part of the haemoglobin structure. The phenotypic classification of thalassaemia patients included in our registry into TDT or NTDT was made by the researchers who follow the patients up, with no defined criteria. TDT manifests in early childhood with severe anaemia or significant complications requiring serial transfusion, while NTDT individuals do not require regular transfusions, although they may require them sporadically. Both groups include patients with HbE/β-thalassaemia and α-thalassaemia (haemoglobin H disease).

### Statistical analysis

The analysis was performed using R 4.3.0 (R Core Team, Vienna, Austria).

Quantitative variables were expressed as median and interquartile range; qualitative variables were reported as absolute and relative frequencies. Death-free survival was studied using the Kaplan–Meier method, after stratifying by the different pathological and clinical variables. Statistical significance was set at p < 0.05.

## Results

A total of 1,874 patients were collected from 80 participating hospitals throughout Spain. We excluded 118 records owing to duplication, leaving a final analysable sample of 1,756 patients.

Among the participating centres, 1 centre contributed more than 400 patients, 1 centre more than 300 patients, 1 centre more than 150 patients, 2 centres between 50 and 100 patients and the remaining 75 centres fewer than 50 patients.

There were 1,317 cases of SCD, 214 cases of thalassaemia and 224 cases with other diagnoses.

### Sickle cell disease

A total of 1,317 patients with SCD were registered (75% of the total sample), with a male/female ratio of 1.09 (52% male, 48% female). The most frequent genotype found was SS (78%), followed by SC (13.5%), Sβ^0^ (4%), Sβ^+^ (4%) and SD (0.5%).

Sixty-seven percent of patients were aged under 18 years, with Spain as the country of birth for most of them (62.5%), followed by Equatorial Guinea (9%).

**Fig. 1:**
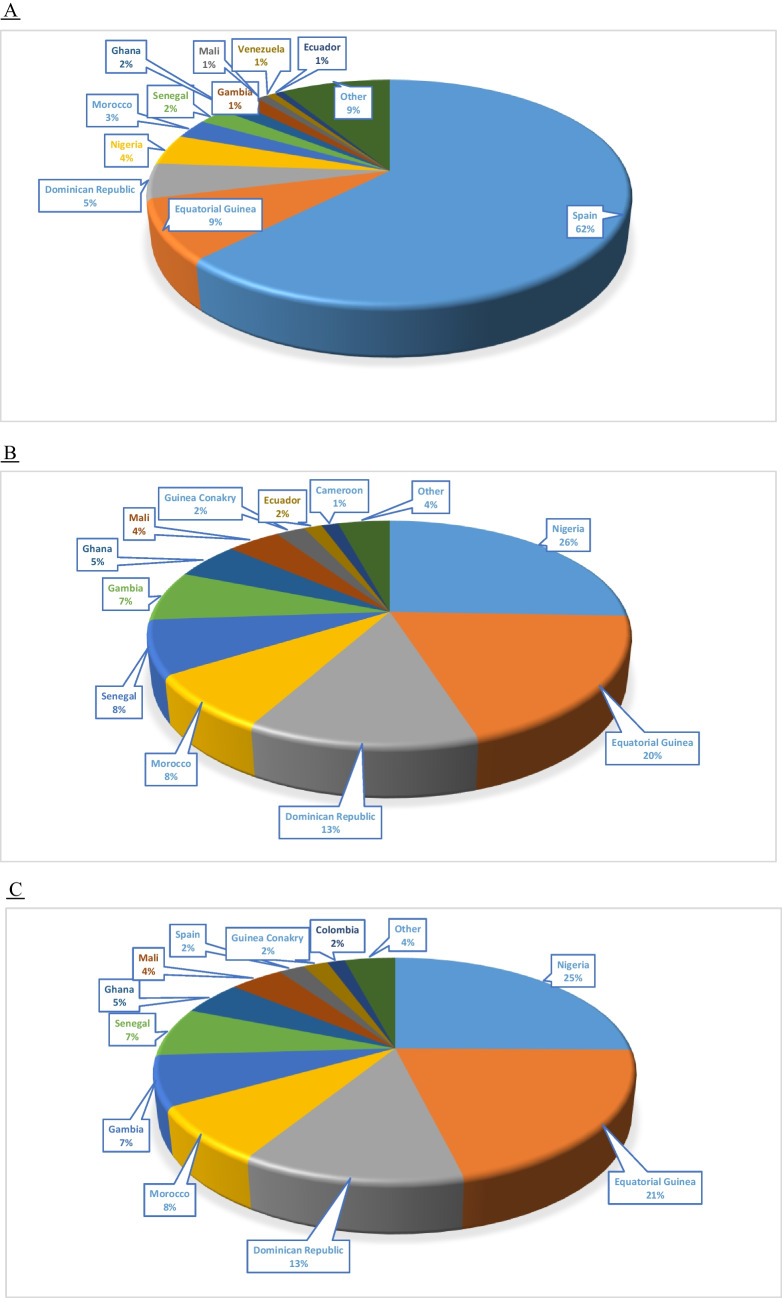
Countries of birth of SCD patients (**A**) and their fathers (**B**) and mothers (**C**). Data are expressed as a percentage

The breakdown of the patients' countries of birth, as well as those of their parents, is shown in Fig. 1.

Diagnosis was based on neonatal screening in 37%, clinical anaemia in 24%, pain episodes in 15% and the family study in 8%. The median age at diagnosis was 2.5 years (0.0–3.0). In addition, 8% had glucose-6-phosphate dehydrogenase deficiency.

The centres with the highest number of patients with SCD were the Gregorio Marañón, Vall d'Hebrón and Sant Joan de Déu hospitals, with 25.6%, 5.4% and 8.9%, respectively, making Madrid and Catalonia the autonomous communities with the highest number of affected persons registered.

The clinical and demographic characteristics are summarised in Table 1.

**Table 1:** Clinical and demographic characteristics of patients included in the REHem-AR. Frequencies are expressed as absolute numbers and, in parentheses, as percentages. Age is expressed as median and, in parentheses, interquartile range. Patients in follow-up correspond to patients who were not classed as deceased or lost to follow-up. SCD, sickle cell disease; HSCT, haemopoietic stem cell transplant; HHA, hereditary haemolytic anaemia; CDA, congenital dyserythropoietic anaemia; CSA, congenital sideroblastic anaemia; DT, transfusion-dependent thalassaemia; NTDT, non-transfusion-dependent thalassaemia; PK, pyruvate kinase; G6PHD, glucose-6-phosphate dehydrogenase

Haemoglobinopathy	SCD	Thalassaemia	Other rare anaemias	
No. of patients	**75% (1.317):** - SS 78% - SC 13.5% - Sβ^0^ 4% - Sβ^+^ 4% - SD 0.5%	**12% (214):** - TDT 54% (115) - NTDT 46% (99)	**13% (224)**	
			*Other Hbpathies and congenital anaemias*	*Enzymopathies*
			43.5% (97): - CC 20.5% (20) - DD 1% (1) - OC 1% (1) - OO 1% (1) - Other Hb 76% (74) 7% (16): - Dyserythropoietic a. 50% (8) - Xerocytosis 37.5% (6) - Sideroblastic a. 12.5% (2)	46% (103): - PK deficiency 19.5% (20) - G6PHD deficiency 80.5% (83)
Male/female ratio	1.09	1.01	1.14	5
Median age at diagnosis	2.5 (0.0–3.0)	1.0 (0.0–3.0)	-	-
Most common reason for diagnosis	Neonatal screening	Anaemia	Anaemia	Anaemia
Deceased	2% (27)	4% (8)	0	0
Lost to follow-up	39% (456)	20% (43)	-	-
HSCT	6.5% (83)	23% (50)	0	1% (1)
COVID-19	9% (119)	55% (118)	-	-

Stroke risk was assessed annually using transcranial Doppler ultrasound (TCD) in 55% of patients with SCD and the SS, Sβ^0^, Sβ^+^ or SD genotype aged 2 to 16 years between 1 January 2019 and 1 June 2023 (the vast majority, annually). TCD was not performed in the other 45% of patients in this age range, in some cases because the patient had received a transplant (16%) and in others because the patient was included in a long-term transfusion programme (29%).

Since our last publication in 2017, an annual average of 13 patients had a pathological outcome on TCD. The highest percentage was in 2018, with a pathological outcome in 20 of the 187 patients (10.5%) aged 2–16 years who underwent TCD in that year.

During the same period, 220 patients (34.5%) with SCD and genotype SS, Sβ^0^, Sβ^+^ or SD underwent brain magnetic resonance imaging, with an abnormality detected in 45% (100 patients). Lacunar infarcts were the most frequent (60%), followed by moyamoya-type vasculopathy (10%). During recent years, 19% had a cerebrovascular accident, and neurocognitive dysfunction was detected in 69%.

**Fig. 2:**
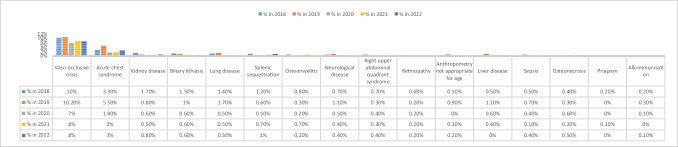
Incidence of the most frequent complications secondary to sickle cell disease (SCD) since our last publication in 2017. Data are expressed as a percentage over the total number of patients with SCD in follow-up that year. Vaso-occlusive crises were defined at the discretion of each investigator (they have been homogeneously defined in the registry subsequent to this analysis). Kidney disease includes proteinuria, kidney failure and nocturnal enuresis. Lung disease includes restrictive disease, obstructive disease (asthma), mixed disease, hypoxaemia and sleep apnoea-hypopnoea syndrome. Neurological complications include cerebrovascular accident (CVA) and neurocognitive abnormalities other than the sequelae of symptomatic CVA. Liver disease includes haemosiderosis or viral hepatitis. *Onset of chronic complications (e.g. nephropathy, biliary lithiasis) during the year in question. Not included if already present

The annual incidence of complications of SCD since our last publication in 2017 is summarised in Fig. 2, according to the total number of patients in follow-up each year.

Regarding treatment, 70.5% (809) of patients with SCD and genotype SS, Sβ^0^, Sβ^+^ or SD started antibiotic prophylaxis with penicillin at some point; this percentage increased to 93.5% in patients aged between 2 months and 5 years of age. Currently, 78.5% of all those who started penicillin continue to receive it (5.5% splenectomised), with an increase in age (28.5% of patients between 5 and 10 years of age, 43.5% of patients between 10 and 20 years of age).

Vitamin D prophylaxis was prescribed at some point for osteopenia in 52% of patients, and 90% were still receiving it at the time of this analysis.

Hydroxyurea was initiated in 686 patients (52%), of whom 81% continue to receive the drug, with a median age at initiation of 5 years (2.0–9.0) and a median duration of 2 years. Excluding patients with SC and Sβ + genotype, the proportion of SCD patients receiving hydroxyurea is 60%.

Ten percent of patients (133) joined a long-term hypertransfusion programme. Of these, 46.5% continue to receive transfusions. The median age of onset was 7 years (4.0–11.0), and the median duration of the programme was 1 year (0.0–2.0).

Chelation therapy was initiated in 7.5% of patients, with deferasirox being the most frequently used drug (93.5%), followed by deferoxamine (15%). The median age at initiation of chelation therapy was 9 years (6.0–14.0), with a median duration of 1 year (0.0–2.0).

A total of 51 patients (4%) underwent splenectomy at a median age of 4 years (2.0–8.0), and 75 patients (6%) underwent cholecystectomy. Ten per cent of the patients underwent another type of surgical intervention, such as adenotonsillectomy or joint replacement. Ten per cent (133 cases) required a central venous catheter (CVC).

Haematopoietic stem cell transplantation (HSCT) was performed in 83 patients (6.5%) at a median age of 7 years (4.0–10.0). Of these, 62% had complete chimerism and 29% had mixed chimerism. Five patients (6%) experienced graft failure. Seven patients (8%) subsequently developed chronic graft-versus-host disease (GvHD).

**Fig. 3:**
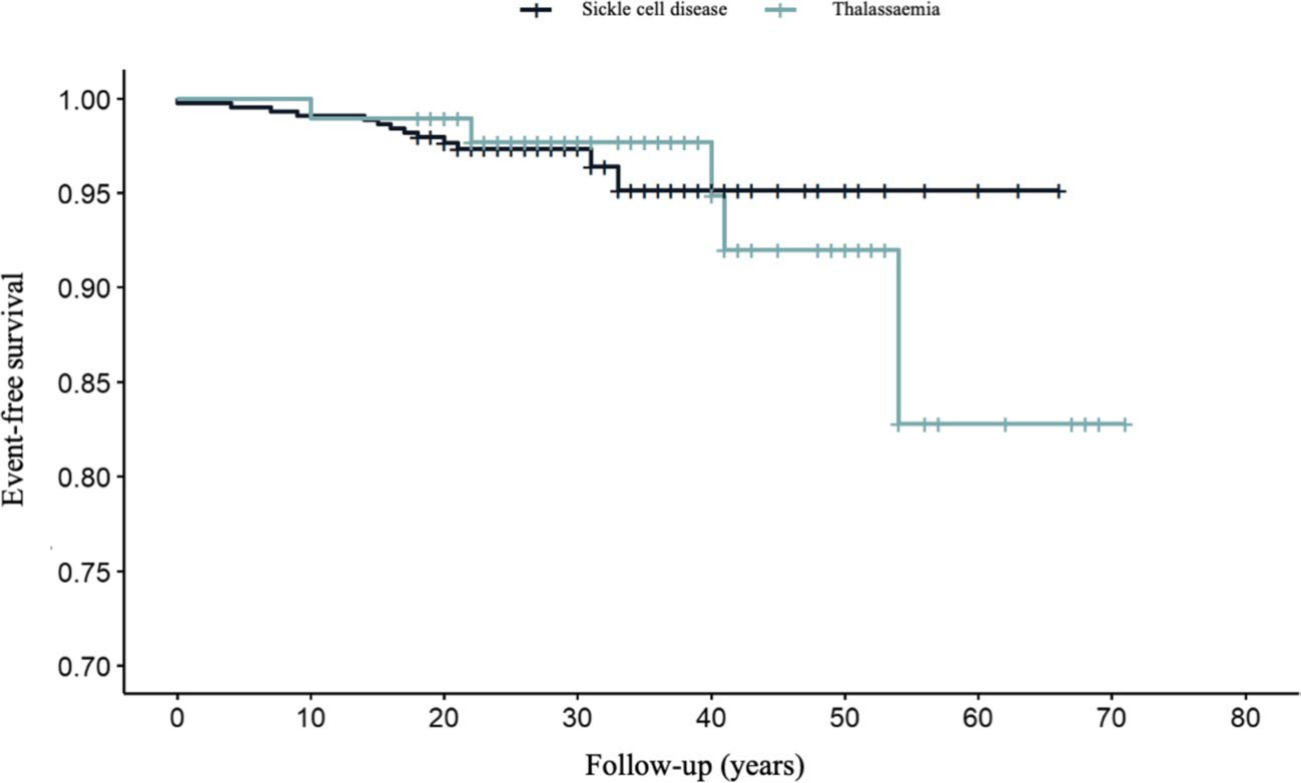
Kaplan–Meier curves. Overall survival in sickle cell disease and thalassaemia

The overall survival (death-free) was 99% (95% CI 98–100%) at 10 years, 97% (95% CI 96–99%) at 30 years and 95% (95% CI 92–99%) at 60 years. Survival curves for both SCD and thalassaemia are shown in Fig. 3.

Thirty-nine percent of the patients in the sample were lost to follow-up owing to emigration to another country (26%), follow-up changed to another centre (42%), and unknown causes (30%).

Twenty-seven patients (2%) died.

As for SARS-CoV-2 infection, a total of 119 patients (9%) were diagnosed with COVID-19. Thirty-eight patients (32%) required admission, and up to 79.5% required empirical intravenous antibiotic therapy. There were no COVID-19–attributable deaths.

### Thalassaemia

The registry included 214 patients with thalassaemia (12% of the total sample), of whom 115 (54%) had TDT and 99 had NTDT. Of the patients with NTDT, 28% had haemoglobin H disease. The male/female ratio was 1.01 (50.5% male, 49.5% female). The centres with the highest number of patients were the Vall d'Hebrón, Virgen del Rocío, La Fe and Gregorio Marañón hospitals, with 25.9%, 12.4%, 11.4% and 10.3%, respectively, making Catalonia, Andalusia, Valencia and Madrid the autonomous communities with the highest number of registered cases of thalassaemia.

**Fig. 4:**
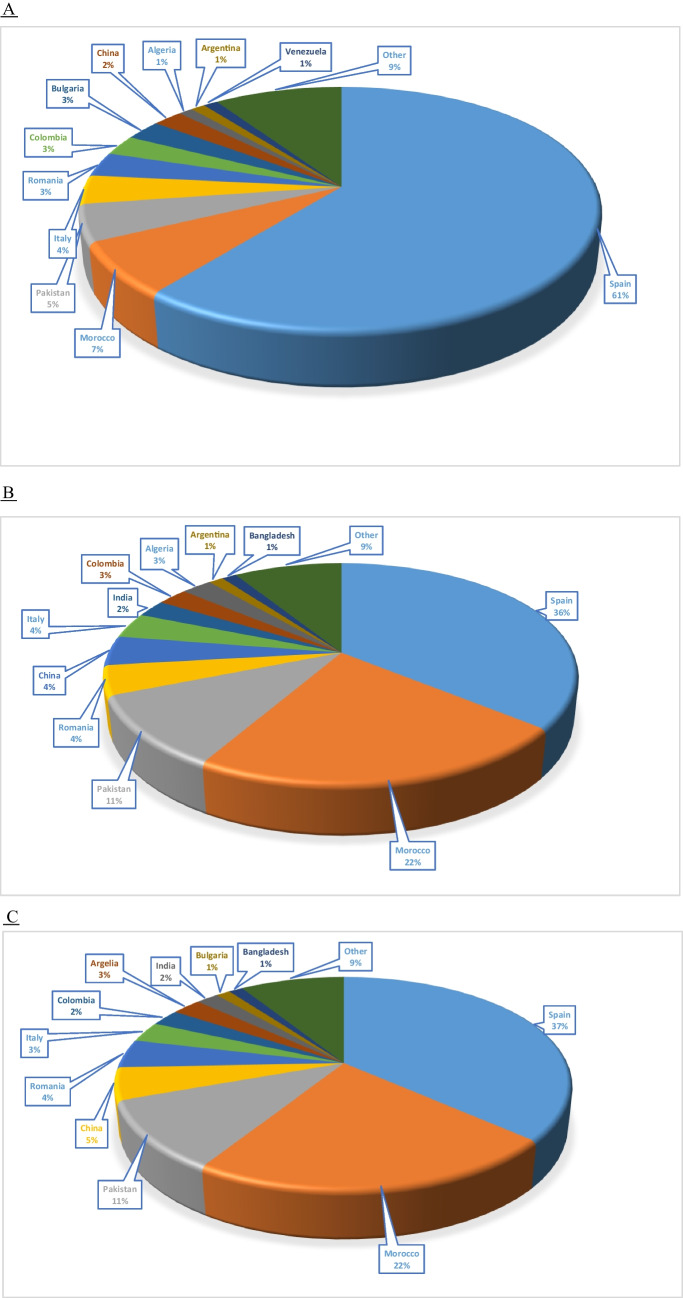
Most frequent countries of birth of thalassaemia patients (D), their fathers (E) and their mothers (F)

Spain was the country of birth for most cases, with 61% of the total, followed by Morocco, with 7%. Paediatric patients (< 18 years) accounted for 47% of cases. The breakdown of the countries of birth of the patients, as well as that of their parents, is shown in Fig. 4.

The main reason for diagnosis was clinical anaemia (71%), followed by an abnormal neonatal screening result (10%) and a family study (8.5%); 10% were diagnosed for other reasons. Considering TDT cases diagnosed in the last 5 years (11 patients), 54.5% were diagnosed following neonatal screening, while among cases diagnosed before 2018, the majority (84.5%) were diagnosed via the anaemia clinic.

Positive hereditary thrombophilia findings were recorded at diagnosis in 4% of cases.

Clinical and demographic characteristics are summarised in Table 1.

In terms of treatment, 90% of patients with TDT without primary parahyperthyroidism received chelation therapy at some point during follow-up, as did 30% of patients with NTDT. In both groups, deferasirox was the most frequently used drug (89%), followed by deferoxamine (46.5%) and deferiprone (23%). The median age at initiation of chelation therapy was 2 years (1.0–3.0), with a median duration of 3.5 years (2.5–5.0).

Thirty-three per cent of patients (71) initiated prophylaxis for osteopenia with vitamin D, and 83% of these patients (59) continued to take it at the time of analysis. Hydroxyurea was associated with standard treatment in 18 patients (8.5%), all of them NTDT. The median age at initiation was 11.5 years (5.0–25.8), with a median duration of 1 year (0.0–1.5). Three patients (16.5%) are still receiving hydroxyurea.

Thirty-two percent (69 patients) required implantation of a CVC, and 15.5% (33 patients) underwent splenectomy at a median age of 10 years (6.0–22.0). Thirteen patients (6%) underwent cholecystectomy.

**Fig. 5:**
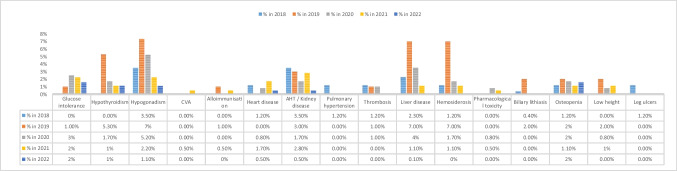
Incidence of the most frequent complications secondary to thalassaemia, annually since 2018. Data are expressed as a percentage. Hepatopathy includes viral hepatitis, haemosiderosis and drug toxicity

The annual incidence of thalassaemia complications since our last publication in 2017 is summarised in Fig. 5, according to the total number of patients registered each year.

Fifty patients (23%) underwent HSCT, at a median age of 6 years (3.0–9.0). Fifty percent had complete chimerism at the time, 16% had mixed chimerism and 8 patients experienced graft rejection. Chronic GvHD occurred in 12% (6 patients).

The overall survival (death-free) was 99% (95% CI 97–100%) at 10 years, 98% (95% CI 95–100%) at 30 years and 83% (95% CI 66–100%) at 60 years. Survival curves for both SCD and thalassaemia are shown in Fig. 3.

Forty-three patients (20%) were lost to follow-up owing to emigration to another country (30%), follow-up changed to another centre (53.5%), and unknown causes (16%). Eight patients (4%) died, with the following causes: 4 related to cardiac hemosiderosis (at 54 years, undated, 11 years and 22 years respectively); 1 due to sepsis, at 4 years old; 1 due to severe liver failure, at 41 years old; 1 due to multifactoral respiratory and cardiac insufficiency, at 41 years; and 1 due to GvHD, at 3 years old.

Regarding COVID-19 data, 118 patients (55%) have presented with the infection since 2020. 19 patients (16%) required admission and only 6.5% received empirical intravenous antibiotic therapy. There were no associated deaths.

### Other rare anaemias

A total of 224 patients (13% of the total sample) were registered with other diagnoses of interest, which are broken down below.

### Other haemoglobinopathies

Haemoglobinopathies other than those previously described were recorded in 43.5% (97 patients), as follows: 20 patients (20.5%) with HbCC, 1 patient with HbDD, 1 patient with HbOC, 1 patient with HbOO and 74 patients (75.5%) with other haemoglobinopathies.

Most of the patients were diagnosed based on abnormalities in the haemoglobinopathy study as part of neonatal bloodspot screening or the finding of anaemia in the blood analysis. Most (86.5%) were born in Spain, as were their parents. The centres with the largest number of patients were the Gregorio Marañón and Vall d'Hebrón hospitals, with 52 and 29 cases, respectively.

#### Enzymopathies

A total of 103 patients (46%) had an enzymopathy: pyruvate kinase (PK) deficiency in 20 patients (19.5%) and glucose-6-phosphate dehydrogenase (G6PHD) deficiency in 83 patients (80.5%). The centres with largest percentages of patients were Vall d'Hebron (29.5%) and Sant Joan de Déu (23.5%).

In both diseases, most cases (65% in PK deficiency and 54% in G6PHD) were diagnosed mainly based on clinical compatibility or anaemia in laboratory tests, followed by family study findings.

Despite the high heterogeneity observed, the country of birth of most patients and their parents was Spain in both cases.

As for the treatment of patients with PK deficiency, up to 45% (9 patients) had received chelation therapy, with deferasirox being, once again, the most commonly used drug. Of these, 44% continue to receive deferasirox. Eight patients (40%) underwent splenectomy. One patient underwent HSCT and did not present GvHD.

#### Other hereditary anaemias

Another type of congenital anaemia was diagnosed in 7% (16 patients): congenital dyserythropoietic anaemia in 8 patients (3 patients with subtype IB, 2 patients with subtype II), xerocytosis in 6 patients and congenital sideroblastic anaemia in 2 patients.

Up to 69% of the cases (11 patients) were diagnosed based on anaemia in the blood analysis. Most (85.7%) were born in Spain, as were their parents.

None of the patients received chelation therapy at the time of the study. One patient with dyserythropoietic anaemia had undergone splenectomy, and none had received HSCT.

## Discussion

**Fig. 6:**
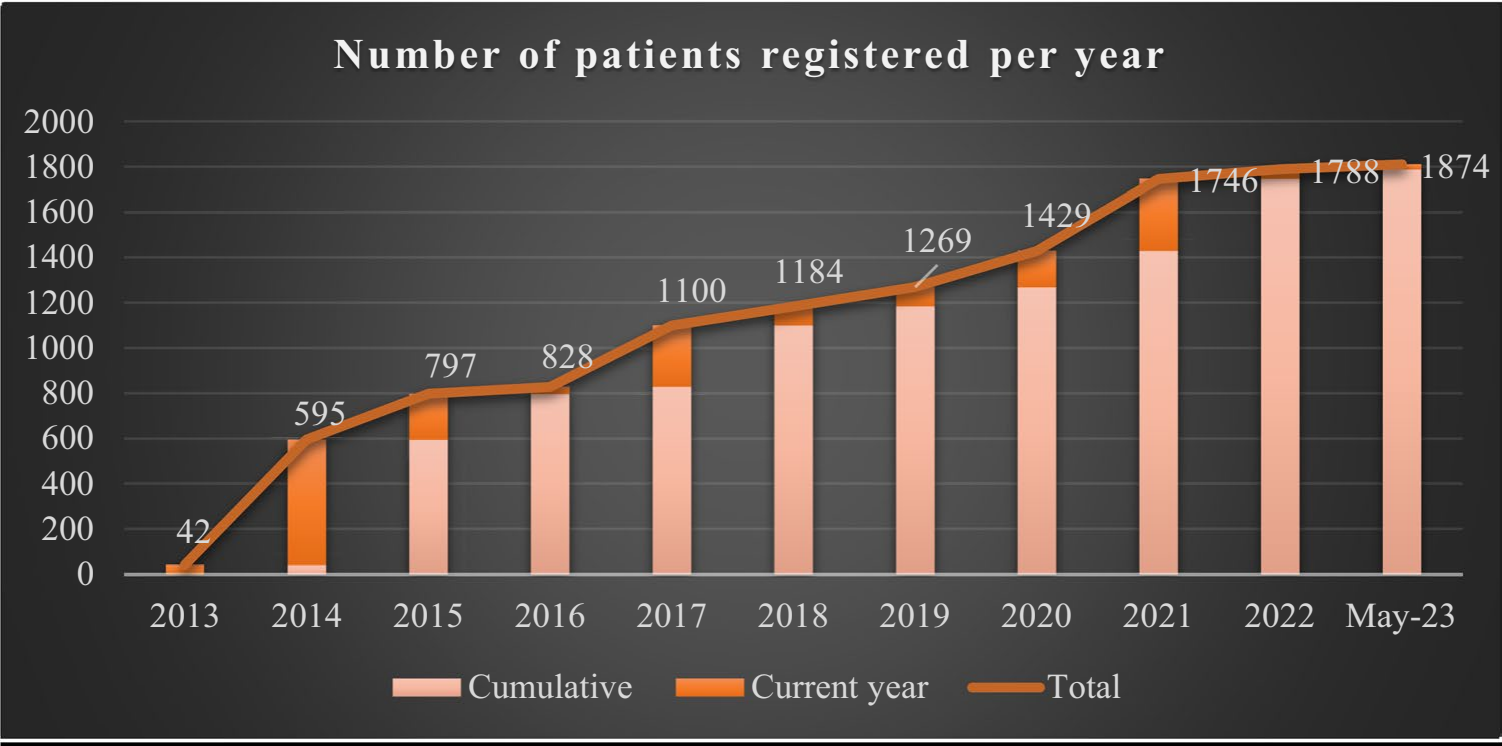
Number of patients registered per year since the registry was created in 2013

The increase in cases in REHem-AR has been progressive in recent years, as can be seen in Fig. 6, with a peak between 2020 and 2021 of more than 300 cases, probably due to a general call for the update of patients made during that year to the hospitals included in the registry, or to an increase in the migratory flow to Spain. An increase in the birth rate seems less plausible, since this decreased in Spain from 7.19% in 2020 to 7.12% in 2021, according to data collected by the National Institute of Statistics (INE). The finding is consistent with a study conducted in the USA, where the demand for contraceptive therapies increased and conception decreased considerably in relation to the COVID-19 [9] pandemic.

In recent decades, migration from areas with a higher prevalence of haemoglobinopathies to Europe has increased. In the specific case of Spain, data from the INE show that in the last 20 years, the number of foreigners from Africa, Central and South America (where many of our SCD patients or their parents come from) and Asia has almost doubled, from approximately 1,700,000 in 2003 to 3,300,000 in 2022; in the specific case of migrants from Africa, the increase has been greater, 2.3 times higher than in 2002 [10, 11]. Consistent with this, most patients' parents are from Nigeria and Equatorial Guinea (in the case of SCD) and Morocco and Pakistan (in the case of thalassaemia), thus supporting the figures reported above for Spain.

In Spain, the number of new diagnoses has increased owing to the progressive implementation of screening in all autonomous communities [12]. In 2021 alone, 68 new cases in the registry corresponded to children diagnosed that year through neonatal bloodspot screening, making it the most frequent means of diagnosis of SCD and other haemoglobinopathies in Spain.

Focusing on patients with SCD, the most frequent complications were vaso-occlusive crisis (10% of patients in 2019, 7% in 2020) followed by acute chest syndrome (5.5% in 2019, 2% in 2020). Consistent with the literature, this finding reflects the complex pathophysiology of the disease, which is largely dependent on microcirculatory occlusion due to the hyperinflammatory state typical of affected patients [13–15].

In the case of thalassaemia, the most frequent diagnostic indicator is clinical anaemia, although the most recent cases of TDT were diagnosed as a result of neonatal bloodspot screening for SCD. Of note is the low incidence of stroke (0.5% in 2021, no cases reported all other years), similar to that found in other series comprising several thousand patients (around 0.25%), and the low rate of alloimmunisation, which is probably related to the usual practice of extensive erythrocyte phenotyping and transfusion based on the ABO, Rh and Kell groups, at least in the large centres that provide the most patients to REHem-AR. Endocrinopathies continue to be the main complication (*Gupta and Aggarwal* report up to 65% prevalence for this type of complication [16]), followed by haemosiderosis and the pathophysiology of the disease itself, despite adequate chelating treatment [17]. Consistent with findings reported elsewhere, we found hypogonadism to be the most prevalent endocrinopathy in recent years [16, 18].

Only a small percentage of thalassaemia patients have ever received hydroxyurea, all of them NTDT, for which some studies have shown a reduction in the frequency of anaemia in specific patients.

Only 60% of patients with SCD and an SS or Sβ^0^ genotype initiated hydroxyurea, a lower number than would be expected based on the recommendations in the guidelines of the Spanish Society of Paediatric Haematology and Oncology (SEHOP, 2019) [8], which favour starting the drug in all children at 9 months of age even if they are asymptomatic, and the recommendations of the Spanish Society of Haematology and Haemotherapy (SEHH, 2021) [19], which favour continuing it into adulthood in patients who were taking it or starting it in those who present symptoms or signs of organ damage.

Of note is the increase in HSCT procedures in SCD compared with the previous publication (3.5% previous vs. 6.5% current). The classic indications are restrictive and limited to specific and severe cases, such as a previous episode of stroke or severe and recurrent acute chest syndrome [8]. However, in recent years, these indications have been revised, given that the potential future severity of the disease is considered a sufficient criterion for transplantation in affected patients. In addition, overall and event-free survival outcomes are significantly higher in patients aged younger than 12–15 years, since transplant-related mortality increases in parallel with age [20–22].

The overall survival of patients with SCD and thalassemia in our study is consistent with data from other European countries (being much lower in countries such as Nigeria, Guinea or Pakistan, where most patients die before 5 years of age) [15, 20], largely influenced by the implementation of penicillin prophylaxis from 2 months of age in the case of SCD, adequate transfusion support and chelation therapy in the case of thalassaemia, and the prevention of associated complications in both cases [23–26].

On January 29, 2020, the WHO reported the first cases of SARS-COV-2 in the Eastern Mediterranean area. The social isolation measures that ensued to prevent transmission of the disease adversely affected the storage and transport chain of blood transfusion networks, which are indispensable for many haemoglobinopathy patients [27].

Along with the decrease in blood supply, an increase in the demand for blood of up to 10–25% was recorded in some centres. To date, our registry has only recorded the percentage of patients with a diagnosis of SCD who have received and are currently receiving a hypertransfusion regimen. We have never recorded the periodicity of transfusions; therefore, it has not been possible to see a change in this area during these years. However, with respect to the latest update of our registry, the number of cases that received a long-term hypertransfusion regimen has not been significantly affected (10% current vs. 8% previous).

Furthermore, it can be assumed that patients with SCD and/or thalassaemia are at greater risk of developing complications secondary to SARS-COV-2 infection [28, 29]. Therefore, most of the patients with SCD in our registry received empirical antibiotic therapy, in line with experience gathered in Great Britain and France [30]. None of the patients in our registry died.

The main limitation of this study is that REHem-AR is not population-based, and the inclusion of patients depends on the health care personnel who volunteer to follow the patients up. Furthermore, REHem-AR arose within the SEHOP and was later extended to include adult patients. This explains the greater participation of specialists caring for minors and the greater weight that minors have in the cohort registered, even though the proportion of adult patients is expected to be greater, since these diseases manifest throughout life (except in the case of primary parahyperthyroidism). Another important limitation continues to be the high rate of loss to follow-up in the registry, due mainly to the transfer of patients back to their country of origin or to a change in follow-up to a hospital not belonging to the REHem-AR network. Follow-up is also hampered by low socioeconomic level and the lack of recognition of the potential severity of the disease in some of our patients.

Moreover, the fact that data are entered into the registry by a multitude of different investigators limits the homogeneity of the response criteria. Some variables had not been well defined at the time of data collection and were defined a posteriori (e.g., vaso-occlusive crisis), and other variables have been added to complement them.

REHem-AR was recently linked to the European registry Rare Anaemia Disorders European Epidemiological Platform (RADeep), which is included in the ENROL study and in the framework of the European network ERN-EuroBloodNet. RADeep started its activity in 2017 with the aim of analyzing the demographics, survival, diagnostic methods, clinical manifestations and treatments of rare anaemias [31] in the European Union. Twelve member states, including Belgium, Germany and Spain, are currently part of the network.

## Conclusion

Since the previous publication in 2019, and following the inclusion of new centres in the registry (currently 80), the number of cases in REHem-AR has increased by more than 500, in parallel with the increase in migration from Africa, Asia and Central and South America.

Vaso-occlusive crisis and acute chest syndrome continue to be the most important complications in SCD, followed by endocrinopathy in patients with thalassaemia. These data are consistent with those described in the literature. The life expectancy of patients with SCD and thalassaemia in our registry is consistent with that of other European countries.

The increase in the performance of HSCT in SCD represents a paradigm shift in the classic indications for these patients, although with the advent of gene therapy, it remains to be seen whether this trend will change in the coming years.

The data reflected in this work go some way to increasing our understanding of haemoglobinopathies and rare anaemias. The availability of new data can help to promote the development of new proposals to improve the diagnosis and treatment of these diseases.

## Declarations

S.P.P. has received speaker or consulting fees from Novartis, Agios, Alexion Pharmaceuticals, Amgen, Pfizer and Vertex Pharmaceuticals.
